# Research update on the association between SFRP5, an anti‐inflammatory adipokine, with obesity, type 2 diabetes mellitus and coronary heart disease

**DOI:** 10.1111/jcmm.15023

**Published:** 2020-01-31

**Authors:** Di Wang, Yaping Zhang, Chengxing Shen

**Affiliations:** ^1^ Department of Cardiology Shanghai Jiao Tong University Affiliated Sixth People's Hospital Shanghai China

**Keywords:** adipokine, anti‐inflammatory, coronary heart disease, obesity, SFRP5, type 2 diabetes mellitus, WNT5A

## Abstract

Secreted frizzled‐related protein 5 (SFRP5), an anti‐inflammatory adipokine secreted by adipocytes, has been demonstrated to exert its anti‐inflammatory effect via antagonizing the non‐canonical wingless‐type family member 5A (WNT5A) signalling pathways. The WNT5A protein, as a potent pro‐inflammatory signalling molecule, is strongly involved in a variety of inflammatory disorders such as obesity, type 2 diabetes mellitus (T2DM) and atherosclerosis. In this review, we systematically outlined the current understanding on the roles of SFRP5 in the pathogenesis of three inflammatory diseases including obesity, T2DM and coronary heart disease (CHD). Our review might stimulate future research using SFRP5 as a promising novel therapeutic target for the treatment of obesity, T2DM and CHD.

## 1 INTRODUCTION

Globally, coronary heart disease (CHD) remains the leading cause of morbidity and mortality.^1^ Atherosclerosis has been regarded as the main underlying mechanism of CHD. The classical doctrine indicates that atherosclerosis is a chronic inflammatory condition characterized by an inflammatory response of the arterial wall (generally speaking, including the tunica intima, media and adventitia) to injuries promoted by risk factors such as obesity, type 2 diabetes mellitus (T2DM) and so on.^2^ It was suggested that low‐grade inflammation stress might contribute to the pathogenesis of atherosclerosis.^3^ More recently, accumulated compelling evidence has delineated the indispensable roles of perivascular adipose tissue in the pathogenesis of atherosclerosis.^4^, ^5^, ^6^, ^7^, ^8^, ^9^, ^10^ In brief, perivascular adipose tissue lying on the outside of adventitia without laminar structures or any organized barrier to separate them^6^ might affect the development of atherosclerosis by releasing pro‐inflammatory adipokines (eg, leptin and resistin) and anti‐inflammatory adipokines (eg, adiponectin).^11^

**Figure 1:**
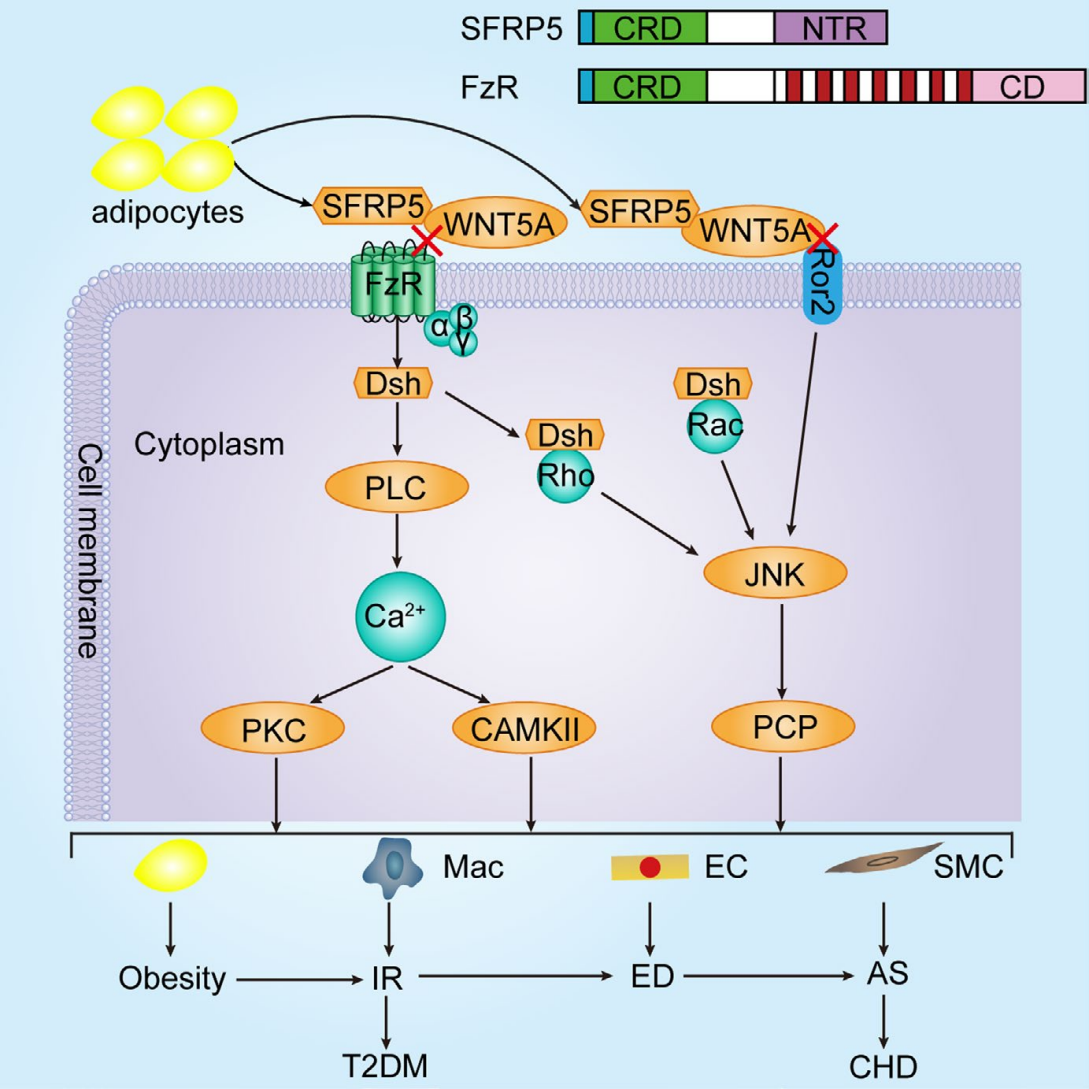
SFRP5 at the crossroad between obesity, T2DM and CHD. As a member of the non‐canonical WNT family of proteins, WNT5A mainly activates WNT5A/Ca^2+^ and WNT5A/JNK signalling pathways. One branch of the non‐canonical signalling pathways involves the activation of small GTPases Rho and Rac. After WNT5A binds to FzR, activated DSH integrates Rho and Rac into it, respectively. Then, the DSH‐Rho and DSH‐Rac complexes activate JNK to regulate the PCP pathway. In addition, WNT5A can also bind to Ror2 to directly activate JNK to mediate the PCP pathway. This signalling branch has been shown to regulate cell orientation. Another branch, when activated, leads to PLC‐mediated increase in intracellular Ca^2+^ levels, which further activates CAMKII and PKC. This signalling branch has been demonstrated to mediate cell proliferation, migration and adhesion. Inflammation plays an important role in this process, which is from obesity, IR and ED to atherosclerosis. As we know, CHD has been regarded as an atherosclerosis‐related disease and the development of T2DM is significantly correlated with insulin resistance. Therefore, we propose that SFRP5, as an anti‐inflammatory adipokine, might link obesity, T2DM to CHD. Abbreviations: AS, atherosclerosis; CAMKII, Ca^2+^/calmodulin‐dependent protein kinase II; CD, cytoplasmic domain; CHD, coronary heart disease; CRD, cysteine‐rich domain; DSH, dishevelled; EC, endothelial cell; ED, endothelial dysfunction; FzR, Frizzled receptor; IR, insulin resistance; JNK, C‐jun N‐terminal kinase; Mac, macrophage; NTR, netrin‐like domain; PCP, planar cell polarity; PKC, protein kinase C; PLC, phospholipase C; Ror2, receptor tyrosine kinase‐like orphan receptor 2; SFRP5, secreted frizzled‐related protein 5; SMC, smooth muscle cell; T2DM, type 2 diabetes mellitus; WNT5A, wingless‐type family member 5A

Secreted frizzled‐related protein 5 (SFRP5), which is a adipokine recently discovered by Ouchi et al in 2010, is highly expressed in white adipose tissue^12^ and could be detected in the circulating plasma.^13^ It has emerged as an endogenous inhibitor of wingless‐type family member 5A (WNT5A) signalling pathways (Figure 1), including non‐canonical WNT5A/Ca^2+^ and WNT5A/c‐jun N‐terminal kinase (JNK) signalling pathways.^14^ The expression of WNT5A protein has been demonstrated in endothelial cells,^15^, ^16^, ^17^, ^18^ smooth muscle cells 19, 20 and macrophages,^21^, ^22^ and the WNT5A protein plays critical roles in a variety of inflammatory disorders such as sepsis,^21^ rheumatoid arthritis,^23^ pulmonary tuberculosis,^24^ psoriasis vulgaris,^25^ obesity,^26^ T2DM,^27^ atherosclerosis 14, 28, 29, 30, 31 and myocardial inflammation secondary to ischaemia/reperfusion injury^32^ or transverse aortic constriction.^33^ As an endogenous inhibitor of WNT5A signalling pathways, SFRP5 has been suggested to play vital roles in obesity,^12^ T2DM^12^ and CHD.^34^

Given obesity and T2DM have been well recognized as two major risk factors of CHD, there must be several inflammatory mediators linking obesity, T2DM to CHD. However, as a novel anti‐inflammatory adipokine, it remains unclear whether SFRP5 could be a member of these inflammatory mediators. This review summarizes the roles of SFRP5 in these three inflammatory disorders, respectively. Furthermore, we discussed how SFRP5 may represent a novel link between obesity, T2DM and CHD.

## 2 ROLES OF SFRP5 IN OBESITY

As a public health issue, obesity, which is characterized by excess of body fat, especially excessive visceral adipose tissue accumulation, is a multifactorial disorder, involving genetics, hormones, diets and environments, and is considered as a state in which chronic low‐grade inflammation occurs within adipose tissue.^35^ Ouchi et al^12^ have observed that in leptin‐deficient (ob/ob) mice, Zucker diabetic fatty mice and wild‐type mice fed a high‐fat, high‐sucrose diet for 24 weeks, obesity contributed to lower levels of SFRP5 expression, higher levels of WNT5A expression and an increase in the ratio of pro‐inflammatory WNT5A to anti‐inflammatory SFRP5. They also measured SFRP5 expression in visceral fat biopsy specimens of obese patients and found that patients with adipose tissue inflammation showed a decrease in SFRP5 transcript expression compared with obese patients without adipose tissue inflammation. These results suggested that regulation of SFRP5 expression is related to obesity.

Recently, multiple clinical studies demonstrated the relationship between anti‐inflammatory SFRP5 and obesity (Table 1). Hu ZP et al^36^ found that circulating plasma levels of SFRP5 were drastically diminished in Chinese obese patients compared with patients with normal bodyweight and plasma SFRP5 concentrations were negatively correlated with body fat parameters, such as body mass index, waist circumference and the waist‐to‐hip ratio. In line with this study, Tan et al^37^ observed that circulating SFRP5 concentrations were significantly lower in obese children, especially in those with metabolic syndrome, and negatively correlated with markers of adiposity, including body mass index and waist circumference. Interestingly, lifestyle intervention gave rise to significant weight loss and up‐regulated SFRP5 expression. Moreover, another clinical study conducted by Hu WJ et al^38^ found that overweight/obese patients had significantly lower SFRP5 levels than lean counterparts, and plasma SFRP5 concentrations were negatively correlated with anthropometric variables, including body mass index, the waist‐to‐hip ratio and per cent body fat. Recently, Akoumianakis et al^39^ demonstrated that circulating WNT5A concentrations were significantly increased in obese patients, accompanied by reduced plasma levels of its antagonist SFRP5. They also found a strong positive association between circulating WNT5A/SFRP5 ratio and body mass index. However, Schulte et al^40^ observed that both circulating serum WNT5a concentrations and circulating serum levels of SFRP5 were elevated in obese patients compared with lean patients. Additionally, Schulte et al^40^ displayed that caloric restriction beneficially increased serum concentrations of anti‐inflammatory SFRP5 in obese patients, while pro‐inflammatory WNT5A levels were not significantly decreased. In contrast, Catalan et al^41^ demonstrated that serum concentrations of WNT5A were decreased in response to surgically induced weight loss, whereas anti‐inflammatory SFRP5 level was not significantly altered. These findings provided a novel regulatory system of chronic low‐grade inflammation in obesity, which could be affected by nutritional intervention or weight loss.

**Table 1:** Clinical studies concerning SFRP5 and WNT5A in obesity, T2DM and CHD

Disorders	SFRP5	WNT5A	IR	References
Obesity	↓↓↓	/	(‐)	Hu ZP et al^36^
	↓↓↓	↑↑↑^a^	(‐)	Tan et al^37^
	↓↓↓	/	(‐)	Hu WJ et al^38^
	↑↑↑^a^	↑↑↑	/	Schulte et al^40^
	↓↓↓^a^	↑↑↑	/	Catalan et al^41^
	↓↓↓	↑↑↑	/	Akoumianakis et al^39^
T2DM	↓↓↓	/	(‐)	Hu ZP et al^36^
	↓↓↓	/	(‐)	Hu WJ et al^38^
	↓↓↓	/	(‐)	Cheng et al^48^
	↓↓↓	/	(‐)	Carstensen‐Kirberg et al^49^
	↑↑↑	/	None	Canivell et al^50^
	↑↑↑	↓↓↓	None	lu et al^51^
CHD	↓↓↓	/	(‐)	Miyoshi et al^34^
	↓↓↓	↑↑↑	/	Akoumianakis et al^39^
STEMI	↑↑↑	/	(‐)	Du et al^55^

Note

↑↑↑ indicates that the SFRP5 levels or WNT5A levels were increased in patients with obesity, T2DM and CHD compared with healthy controls, respectively. ↓↓↓ indicates that the SFRP5 levels or WNT5A levels were decreased in patients with obesity, T2DM and CHD compared with healthy controls, respectively. (‐) indicates that the SFRP5 levels were negatively correlated with IR. None indicates that there were no relationships between the SFRP5 levels and IR. / indicates that the data were not mentioned in this study.

Abbreviations: CHD, coronary heart disease; IR, insulin resistance; SFRP5, secreted frizzled‐related protein 5; STEMI, ST‐segment elevation myocardial infarction; T2DM, type 2 diabetes mellitus; WNT5A, wingless‐type family member 5A.

a Indicates that there was no statistical significance.

As mentioned above, pro‐inflammatory WNT5A and anti‐inflammatory SFRP5 both play pivotal roles in the development of obesity. Therefore, we propose that SFRP5 may exert a protective role in the pathogenesis of adipose tissue inflammation and obesity via non‐canonical WNT5A signalling pathway. Importantly, the specific mechanisms remain to be elucidated through a series of basic studies.

## 3 SFRP5 IN THE PATHOGENESIS OF T2DM

Insulin resistance has been identified as one of the important characteristics of T2DM and also been considered as an indispensable factor underlying the pathogenesis of T2DM, which usually precedes the onset of this disease.^42^ It has been demonstrated that the development of insulin resistance is linked to a macrophage‐mediated inflammation of adipose tissue^43^, ^44^, ^45^ and is associated with chronic low‐grade inflammation.^46^ Therefore, it is boldly speculated that SFRP5, which is a novel anti‐inflammatory adipokine and also an endogenous inhibitor of WNT5A signalling pathways, might prevent macrophage‐mediated inflammation of adipose tissue by antagonizing the WNT5A protein to improve the insulin sensitivity, consequently playing a protective role in the pathogenesis of T2DM. Intriguingly, a study conducted by Ouchi et al^12^ was consistent with this hypothesis. They observed that SFRP5‐deficient (SFRP5^−/−^) mice fed a high‐fat diet showed greater adipose tissue inflammation mediated by macrophage and insulin resistance compared with wild‐type mice due to unrestrained WNT5A activity. On the contrary, two weeks after intravenously administered Ad‐SFRP5, insulin sensitivity was significantly improved. Interestingly, in vitro, up‐regulation of SFRP5 expression in 3T3‐L1 adipocytes prevented the inflammatory, insulin‐resistant state by blocking WNT5A activity. Similarly, Lv et al^47^ found that overexpression and over‐secretion of SFRP5 in 3T3‐L1 adipocytes may be one key mechanism by which rosiglitazone and metformin improve insulin sensitivity.

Additionally, multiple human studies have demonstrated that SFRP5 plays a role in the pathogenesis of T2DM (Table 1). Hu ZP et al^36^ found that circulating plasma concentrations of SFRP5 were decreased in T2DM patients compared with healthy controls and they were negatively correlated with homeostasis model assessment of insulin resistance, suggesting that SFRP5 might exert a protective effect on the mechanism of T2DM. Similar results were observed by clinical studies conducted by Hu WJ et al,^38^ Cheng et al^48^ and Carstensen‐Kirberg et al,^49^ and all indicated a possible role for SFRP5 as a protective factor in the pathogenesis of T2DM. In contrast, Canivell et al^50^ observed that circulating plasma levels of SFRP5 were augmented in patients with T2DM compared with prediabetic patients and healthy controls. These findings are consistent with another study conducted by Lu et. al,^51^ who found that circulating plasma SFRP5 concentrations were also elevated but circulating plasma levels of WNT5A were diminished in T2DM patients compared with non‐diabetic patients. However, there were no relationships between circulating plasma SFRP5 levels and insulin resistance based on results from these two studies.

Collectively, although the protective roles of SFRP5 in the pathogenesis of T2DM have been explored both in animal and cell studies, controversial results were found in clinical studies. Thus, the relationships between SFRP5 and T2DM in human remain to be explored. Therefore, further well‐designed studies with larger sample size and more ethnic groups with respect to the roles of SFRP5 in the pathogenesis of T2DM should urgently be considered.

## 4 SFRP5 CONTRIBUTING TO CHD

Atherosclerosis is the basic pathological foundation of CHD. Multiple mechanisms are linked to the pathogenesis of atherosclerosis, including endothelial dysfunction, which is characterized by an imbalance between endothelium‐dependent vasorelaxation and vasoconstriction,^52^ the migration and proliferation of vascular smooth muscle cells.^53^, ^54^ Breton‐Romero et al^17^ observed that in aortic endothelial cells derived from patients with T2DM, WNT5A induced impairment of endothelial nitric oxide synthase activation and nitric oxide production was reversed by WNT5A and JNK inhibition (Box5 and SP600125). Interestingly, a recent study^18^ published in *Arteriosclerosis, thrombosis and vascular biology *provided the first direct evidence that SFRP5 exerted its restorative effect on the WNT5A‐induced endothelial dysfunction through the inhibition of the WNT5A/JNK pathway and the up‐regulation of endothelial nitric oxide production, which suggests a possible role of SFRP5 as a protective factor in the development atherosclerosis under conditions of metabolic dysfunction. Similarly, Akoumianakis et al^39^ demonstrated that WNT5A could increase vascular oxidative stress and reduce nitric oxide bioavailability in human vessels, which can be restored by SFRP5. Intriguingly, they also observed that WNT5A increased the migration of human vascular smooth muscle cells without affecting their proliferation, an effect reversed by SFRP5. These findings identified SFRP5/WNT5A signalling pathway as a link between obesity and atherosclerosis. Furthermore, Nakamura et al^32^ found that compared with wild‐type C57BL/6 mice, SFRP5^−/−^ mice showed larger infarct sizes, greater infiltration of WNT5A‐positive macrophages and inflammatory cytokine and chemokine gene expression in the ischaemic lesions, increased cardiomyocytes apoptosis and decreased cardiac function following ischaemia/reperfusion injury. And in vitro, the WNT5A protein promoted JNK activation and augmented inflammatory gene expression in bone marrow‐derived macrophages, while treatment with SFRP5 could inhibit these effects. From these findings, we can conclude that SFRP5, as a cardioprotective adipokine, could attenuate cardiac inflammation and protect the heart from ischaemia/reperfusion injury possibly via WNT5A/JNK signalling pathway.

As for clinical studies (Table 1), Miyoshi et al^34^ carried out one clinical study including 185 patients, who were divided into CHD group and non‐CHD group and circulating serum concentrations of SFRP5 were measured. And these investigators for the first time observed that lower serum levels of SFRP5 were significantly related to CHD and the severity of CHD was negatively associated with serum SFRP5 levels, suggesting the link between SFRP5 levels and CHD. But to our regret, they did not measure circulating serum WNT5A levels. Fortunately, a case‐control study conducted by Akoumianakis et al^39^ complemented this work. They found that patients with CHD had higher circulating WNT5A levels, lower circulating SFRP5 levels and a circulating plasma WNT5A/SFRP5 ratio compared with patients without CHD. And the presence of CHD was associated with plasma WNT5A (positive) concentrations, plasma SFRP5 (negative) concentrations and plasma WNT5A/SFRP5 ratio (positive) independently of traditional risk factors (hypertension, hyperlipidemia, diabetes and smoking). Interestingly, they observed that circulating WNT5A levels were independently‐positively associated with calcified coronary plaque progression and new‐onset coronary calcification. This promising result further highlighted the role of SFRP5 and WNT5A in CHD. Besides, SFRP5 may be a promising therapeutic target in acute ST‐segment elevation myocardial infarction (STEMI). Recent study conducted by Du et al^55^ found that serum SFRP5 levels were significantly increased during the early phase of acute STEMI but attenuated over time. Interestingly, high serum SFRP5 levels were significantly correlated with improving cardiac function at an early phase (3 months) after primary percutaneous coronary intervention. Similarly, the serum WNT5A levels were not measured.

However, to date, human studies showing the relationships between SFRP5 and CHD are still scanty and direct roles of SFRP5 in the pathogenesis of atherosclerosis and myocardial infarction hitherto remain obscure. Thus, more clinical and basic studies should be performed urgently.

## 5 SFRP5 AT THE CROSSROAD BETWEEN OBESITY, T2DM AND CHD

There are multiple papers focusing on the crucial roles of adipokines in constituting a critical link between the obesity, T2DM and atherosclerosis or cardiovascular diseases.^11^, ^56^, ^57^ SFRP5, as a novel anti‐inflammatory adipokine, might be at the crossroad between obesity, T2DM and CHD (Figure 1). It is known that obesity, especially the central obesity, mainly participates in the formation and development of insulin resistance by affecting the sensitivity of insulin. Moreover, it has been demonstrated that improving endothelial function could ameliorate insulin resistance, whereas improving insulin sensitivity could ameliorate endothelial dysfunction, which suggesting the reciprocal relationships between insulin resistance and endothelial dysfunction.^58^ Endothelial dysfunction has been identified as the primary and a crucial step of the development of atherosclerosis.^52^, ^59^, ^60^ In addition, inflammation plays an important role in this process, which is also the common disease feature of obesity, insulin resistance and endothelial dysfunction. Therefore, we propose that SFRP5, as an anti‐inflammatory adipokine, might link obesity, T2DM to CHD.

## 6 CONCLUSIONS AND PERSPECTIVES

In this review, we summarized the indispensable roles of SFRP5, a novel anti‐inflammatory adipokine, in the pathogenesis of these three inflammatory disorders, namely obesity, T2DM and CHD. Taken together, we propose that SFRP5 binds to WNT5A and blocks the non‐canonical WNT5A signalling pathways, consequently reducing chronic inflammation and exerting its protective effect on reducing/retarding obesity, insulin resistance and atherosclerosis. SFRP5, as an anti‐inflammatory adipokine, might link obesity, T2DM‐CHD. Thereby, this hypothesis might be of clinical relevance since SFRP5 can be a promising candidate for future treatments of these three inflammatory disorders. Despite ongoing and available research, further studies are required to investigate whether WNT5A non‐canonical signalling pathways are the only one to participate in the development of these three inflammatory disorders or not. This is because WNT5A mainly acts on the non‐canonical signalling pathways; however, it can also activate the canonical β‐catenin signalling pathway in virtue of the receptor availability.^14^ In summary, inflammation is the common disease feature of these three inflammatory disorders—obesity, T2DM and CHD, which makes it possible for SFRP5 to be at the crossroad between obesity, T2DM and CHD. Further exploration of the biological functions of SFRP5 may pave the way for it to serve as a potential novel treatment option for obesity, T2DM and CHD.

## CONFLICT OF INTEREST

The authors confirm that there are no conflicts of interest.

## Data Availability

The data that support the findings of this study are available from the corresponding author upon reasonable request.
